# Tryptophan-induced effects on the behavior and physiology of aging in tryptophan hydroxylase-2 heterozygous mice C57BL/6N

**DOI:** 10.14202/vetworld.2025.296-310

**Published:** 2025-02-13

**Authors:** Anastasiya Kibitkina, Ekaterina Vasilevskaya, Galina Tolmacheva, Elena Kotenkova, Ekaterina Polishchuk, Viktoriya Pchelkina, Sergey Karabanov, Liliya Fedulova

**Affiliations:** 1 Department of Experimental Clinic and Research Laboratory for Bioactive Substances of Animal Origin, V.M. Gorbatov Federal Research Center for Food Systems, Moscow, Russia; 2 Center for Genomic Technology and Bioinformatics, Moscow Institute of Physics and Technology (National Research University), Dolgoprudny, Russia

**Keywords:** aging, behavioral tests, lipid metabolism, neuro-nutrition, neuropsychiatry, serotonin, tryptophan hydroxylase-2 heterozygosity, tryptophan

## Abstract

**Background and Aim:**

Tryptophan (Trp), a precursor of serotonin, plays a critical role in cognitive and emotional processes. Its metabolism through serotonin and kynurenine pathways impacts neuropsychiatric functions and lipid metabolism. This study investigates Trp’s effects on the behavioral, physiological, and molecular parameters of aging female wild-type (WT) and heterozygous tryptophan hydroxylase-2 (HET) mice.

**Materials and Methods:**

A 68-day experiment was conducted on 13-month-old WT and HET mice. Groups received either distilled water or Trp supplementation (400 mg/kg/day). Behavioral tests (Open Field, Elevated Zero Maze, Forced Swim, and Extrapolation Escape Task) assessed locomotion, anxiety, and cognition. Physiological assessments included body composition through NMR relaxometry, lipid histology, serotonin content in the brain (ELISA), and serotonergic gene expression (RT-PCR). Blood biochemistry and organ weights were also analyzed.

**Results:**

Trp supplementation reduced growth rates and adipose tissue while increasing muscle mass in both genotypes, more markedly in HET mice. Behavioral tests revealed a decrease in anxiety and enhanced cognitive performance in HET+Trp mice but an increase in immobility. Trp increased brain serotonin content in HET mice and altered serotonergic gene expression. Histological studies showed hepatoprotective effects in HET+Trp mice, reducing liver lipid infiltration compared to WT+Trp mice.

**Conclusion:**

Trp exhibited genotype-specific effects, with HET mice showing anabolic, hepatoprotective, and neuropsychiatric changes. These findings highlight Trp’s potential in neuro-nutrition for conditions like depression and cognitive decline. Further studies are needed to explore Trp’s metabolic pathways and their implications for personalized dietary interventions.

## INTRODUCTION

There is no doubt that nutrition can affect the psycho-emotional state of humans [1]. In this regard, the direction of neurodietology is actively developing, and research is being conducted on manipulating the composition of diets and the alimentary prevention of diseases of the nervous system for the treatment of neurological and psychological diseases [2].

Essential and non-essential amino acids are necessary for the functioning of the central nervous system. Tryptophan (Trp), tyrosine, histidine, and arginine are used by the brain to synthesize various neurotransmitters and neuromodulators, act as neuromediators, and directly affect cognitive, emotional, and motor processes [3–6]. Trp is the most interesting and studied essential amino acid because it is critical for protein synthesis in the body and is the sole precursor of the neurotransmitter serotonin (5-hydroxytryptamine [5-HT]). Trp is a component of both plant and animal proteins. The highest amounts of Trp are found in hard cheeses, cottage cheese, milk, yogurt, fish, meat, mushrooms, oats, dates, peanuts, sesame seeds, pine nuts, and soybeans. Trp is degraded in the organism in two ways: Serotonin-melatonin and indole-kynurenine-niacin pathways. The 5-HT pathway is regulated by a cascade of interrelated reactions in the brain, and the rate of neurotransmitter production is limited by the enzyme tryptophan hydroxylase-1 (TPH1) and tryptophan hydroxylase-2 (TPH2) [7].

Trp catabolism through the second pathway leads to the formation of kynurenine and its metabolites, which directly and indirectly affect various classical neurotransmitter systems. A number of studies [8–11] have focused on the participation of Trp metabolites in anabolic processes, interorgan communications, and emotional and behavioral reactions.

Considered the transformation of Trp to acetyl-coenzyme A, which then turns into quinolinic acid and ultimately into nicotinic acid, which plays an important role in lipid metabolism by lowering low-density lipoprotein cholesterol and increasing high-density lipoprotein levels [8, 9]. Kynurenines, which easily penetrate the blood-brain barrier, are involved in several processes occurring in living systems [10]. In particular, it shows the role of variations in kynurenine metabolites in the pathogenesis of mood disorders [11], whereas the 5-HT and indole pathways were suppressed. Articles about the serotonergic metabolism of Trp have conflicting data on its effect on neuropsychiatric disorders [12–16].

Some studies have shown decreased 5-HT levels in patients and animals during depression [16–18]. At the same time, with a decrease in Trp intake with food, a decrease in mood is observed only in individuals who previously suffered from depression [19]. Acute Trp depletion had no significant effect on subjects without a history of depressive episodes. This appears to be attributable to altered 5-HT pathways in patients prone to depressive disorder compared with healthy individuals who do not. Thus, the study of Trp and its metabolites for use in neurodietology, particularly in patients prone to depressive disorder, is relevant.

**Figure 1:**
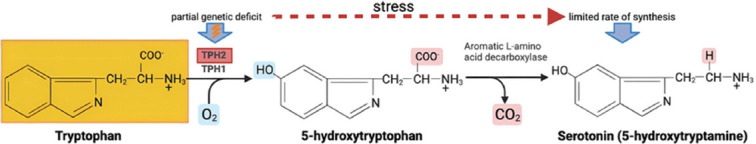
Changes in 5-hydroxytryptamine synthesis in heterozygous tryptophan hydroxylase-2 mice.

Animal models with mutations in genes involved in the transmission of 5-HT and the formation of serotonergic neurons are promising for studying the effects of Trp and its metabolites on neuropsychiatric disorders. A highly accurate model is TPH2 knockout mice. Dysfunctional TPH2 alters 5-HT biosynthesis [20, 21], leading to 5-HT deficiency (Figure 1) and indirectly affecting behavior [22]. Homozygous TPH2 mice are prone to depression and usually show profound behavioral and neurological impairments, whereas stress-induced heterozygous TPH2 (HET) mice show cognitive impairment and signs of obsessive-compulsive disorder [23, 24].

In recent years, the study of Trp has only been gaining momentum; however, previous studies [25, 26] are largely limited by a one-way study of individual pathway-specific metabolites, with only stressed animals being studied, mostly young males as model animals with progressive disease. These studies examined the effect of Trp in aging female mice, as women are more at risk of developing depression and anxiety disorders. At the same time, we were interested in determining how HET mice, endophenotypically normal but genetically susceptible to depression, respond to Trp administration during aging.

In our study, we will consider how the prolonged administration of Trp affects the behavioral, cognitive, and physiological parameters of aging female mice heterozygous for the *TPH2* gene.

## MATERIALS AND METHODS

### Ethical approval

The procedures followed were in accordance with the standards outlined in Directive 2010/63/EU of the European Parliament and the European Union Council for Protection of Animals used for scientific purposes. Research work on animals was carried out in accordance with the NC3R ARRIVE guidelines for *in vivo* experiments. The study was approved by the bioethical commission of the V.M. Gorbatov Federal Research Center for Food Systems of the Russian Academy of Sciences (protocol #01/2022, dated March 14, 2022).

### Study period and location

The study was conducted from July to August 2022 in the Department of Experimental Clinic and Research Laboratory for Bioactive Substances of Animal Origin, V.M. Gorbatov Federal Research Center for Food Systems.

### Animals and experimental groups

The parent strain of HET mice containing Cre-excipient loxP sequences targeting exon 5 was obtained on a mixed genetic background of 129 Sv and C57BL/620.28 and crossed with a pure genetic background of C57BL/6N mice at the laboratory of Dr. Lesch at the Division of Molecular Psychiatry, Center of Mental Health, University of Würzburg in May 2019 (Würzburg, Germany). The studied strain of mice was obtained from harem breeding of HET knockout mice with C57BL/6N mice [27] in V.M. Gorbatov Federal Research Center for Food Systems (Moscow, Russia).

This study was performed for 68 days on 26 wild-type (WT) and HET female mice; all mice were aged 13 months and weighed 32.2 ± 1.7 g.

The mice were divided into groups before the start of the experiment:

- Group 1: Female WT (+/+) (n = 6), who were daily intragastrically treated with distilled water in a volume of 0.2 mL;
- Group 2: Female WT + Trp (+/+) (n = 6), who were daily intragastrically treated with Trp (Global Healthcare, Russian Federation) at a dose of 400 mg/kg dissolved in 0.2 mL of water [28];
- Group 3: Female HET (±) (n = 7), which were daily intragastrically treated with distilled water in a volume of 0.2 mL;
- Group 4: Female HET + Trp (±) (n = 7), who were daily intragastrically treated with Trp at a dose of 400 mg/kg, dissolved in 0.2 mL water.

Animals were kept in groups of 2–3 individuals in ventilated Bio A.S. cages (Vent II, EHRET, Germany) type 2L (350 × 200 × 140 mm). The conditions for keeping animals were standardized: temperature: (20°C ± 3°C), humidity: (35% ± 2%), supply and exhaust ventilation: (95 m^3^/h ± 5 m^3^/h), day/night lighting mode from 6.00 to 18.00/from 18.00 to 6.00. When using hygienic bedding for laboratory animals, Lignocel BK 8-15/LIGNOCEL (J. Rettenmaier and Sohne GmbH, Germany). Throughout the experiment, the animals consumed complete feed (E. Merck KGaA, Darmstadt, Germany) and water for drinking *ad libitum* at the EMD MilliporeRiOs™ 50 water treatment plant (MercMillipore, Germany). The water was mineralized by adding mineral salts (314–382 mg/L: hydrocarbonates - 144–180, sulfates - <1, chlorides - 60–76, calcium - 6, magnesium - 3, sodium - 50–58, potassium - 50–58).

Complete feed (Laboratorkorm, Russia) contained wheat (40%), barley (26%), soybean meal (≤50% crude protein), fish meal (≤60% crude protein), wheat bran (18.0%), sunflower oil (5%), vitamin and mineral premix, and common salt (0.5%) (Table 1).

**Table 1:** Composition of complete feed for mice.

Component	Content, %	Component	Content (per kg)
Macronutrients		Vitamins	
Crude protein	19.0	A	5000 U
Crude fat	5.0	D_3_	500 U
Crude fiber	4.0	E	30.00 mg
Crude ash	9.0	B_1_	2.24 mg
Starch	35.0	B_2_	1.20 mg
Sugar	5.5	B_3_	6.00 mg
Extractives that do not contain nitrogen	52.0	B_5_	22.15 mg
		B_6_	2.33 mg
Minerals		B_7_	6.60 mg
Calcium	1.8	B_c_	10.00 mg
Phosphorus	1.1	B_12_	10.00 mg
Sodium	0.1	K_3_	0.30 mg
Magnesium	0.2	Microelements	
Potassium	0.7	Iodine	0.24 mg
Amino acids		Iron	18.00 mg
Lysine	1.1	>Manganese	9.00 mg
Methionine + cystine	0.7	Zinc	22.50 mg
		Copper	2.40 mg
Tryptophan	0.04	Selenium	0.06 mg
		Cobalt	0.18 mg

### Body composition analysis and body indexes

Mice were weighed to determine the Growth rate using a DX-2000 scale (A&D Company ltd., Japan), and body length was measured using a caliper (Harden, China) to determine the growth rate as follows [29]:

Rg = (W2−W1)/(t2−t1),

where W1 is the body weight of mice on day 0 (g); and

W2 is the body weight of mice on day 68 (g);

t1 is the starting point of time (day 0);

t2 is the end point of time (day 68).

The Lee index was calculated using the following equations [30]:

Lee index = body weight (g)^(1/3)^/(NAL [cm]) × 1000

where NAL is the distance between the nasal passage and the anus of the animal.

The relative content of fat and muscle tissue in mice was measured using a Minispec MQ LF110 nuclear magnetic resonance (NMR) relaxometer (Bruker, Germany) on days 0, 21, 35, 49, and 65. Animals were preliminarily (for 3 days before distribution of groups) adapted to fixation in a red plastic restrainer (50 mm in diameter) and to placement in the NMR relaxometer. The relaxometer was calibrated according to the protocol suggested in the device manual. Before testing, quality control checks of internal stresses, temperature, magnets, and NMR parameters were performed using a standard provided by the manufacturer. For the measurement, mice were placed in a restraint and were kept immobile. The latch was then lowered into the chamber of the device, and the scan took 2 min.

### Anxiety-like behavior and exploratory locomotion

Tests were performed on days 0 and 67 of the study. Motor activity was assessed in the Open Field test (OpenScience, Russia), which is a square area 50 × 50 cm in size with opaque sides 30 cm high. The mouse was placed in the center of the arena, and the testing time was 3 min. The following activities were recorded: distance traveled (m) and number of freeze episodes [31].

Anxiety behavior was assessed in the Elevated Zero Maze test, which consisted of an elevated gray ring platform (diameter 50 cm) with two adjacent open and two closed compartments (walking lane width 5.5 cm) of the same length (30 cm). The animals were placed in one of the closed compartments, and the time spent in the closed compartments was recorded for 6 min [32].

Depressive-like behavior was assessed using the forced swim test. A transparent cylinder (diameter 17 cm, height 18 cm Porsolt) filled with warm water (24 ± 1°C) was used for the test. The latent period and the immobilization duration were recorded by visual observation for 4 min. The absence of any directed movements of the head and body of the animals was considered the immobilization behavior [33].

The “Extrapolation escape task” test was performed to identify mice’s cognitive functions. The experimental setup was a cylindrical container (diameter 35 cm, height 40 cm) filled with water up to 12 cm, with a ladder 16 cm high lowered into the water. The animals were placed in water, and the time at which a mouse climbed the upper rung of the ladder was recorded [34].

Video recording of tests was performed using a Nikon D5600 18-55 VR kit camera (Nikon, Thailand), and records were processed using ToxTrac software (Umea, Sweden) and RealTimer (OpenScience) [35].

### Euthanasia and sampling

On the 68^th^ day of the experiment, the animals were anesthetized using Xylosin (Interchemie, Estonia) at a dose of 5 mg/kg and Zoletil (TDVet, Spain) at a dose of 20 mg/kg. Blood was taken from the right atrium into tubes with ethylenediaminetetraacetic acid (Aquisel, Spain) to analyze whole blood and plasma parameters. Plasma was obtained through centrifugation in a CM-6M centrifuge (ELMI, Latvia) for 8 min at 2260 x *g*. Plasma was frozen and stored at a temperature of 41°C ± 1°C.

After blood sampling, transcardial perfusion with 0.9% saline was performed, next the mice were euthanized by decapitation.

Next, the brain, liver, left kidney, visceral fat, muscle (*Musculus soleus*), and spleen were weighed on an electronic balance (Acculab Vicon, Canada) to determine the relative mass of the internal organs.

Samples for histological studies: the right medial lobe of the liver (Lobus hepatis dexter medialis) and *M. soleus* of the right limb were taken within 10 min after decapitation.

After cortex separation, brain samples were stored at - (80 ± 1°C) until assayed.

### Blood analysis

In whole blood, the content of lymphocytes (LYM), granulocytes (GRA), and monocytes (MON) was determined by detecting cell size and granularity on a Guava Easy Cyte flow cytometer (Merck Millipore, Germany). The leukocyte content was determined using the following formula: WBC = LYM + GRA + MON.

The following biochemical parameters were determined in the blood plasma: Sodium, glucose, total protein, albumin, creatinine, urea, total cholesterol, triglycerides, high-density lipoprotein (HDL) and low-density lipoprotein (LDL), aspartate aminotransferase (AST), and alanine aminotransferase (ALT) levels on the BioChem FC-360 automatic biochemical analyzer (HTI, USA) using reagent kits (HTI).

The level of cholesterol metabolism was assessed using the atherogenic index (AI) expressed in relative units (r.u.) according to the following formula [36]:

AI = (Total cholesterol−HDL)/HDL

The de Ritis coefficient was calculated as the ratio of AST to ALT activity according to the following formula [36]

de Ritis coefficient = AST/ALT.

### Histological study

Samples were stained with Oil Red O (ORO) (Sigma-Aldrich, Germany) according to themanufacturer’s protocol [37]. Sections of 10 µm thick were obtained on a Microm HM-525 cryostat (Thermo Scientific, USA) and mounted on Menzel-Glaser glasses (Thermo Scientific), washed with 60% isopropanol, incubated in 0.5% ORO solution (Sigma-Aldrich) for 10 min, and washed them again with 60% isopropanol and distilled water. The histological preparations and photographs were studied on an AxioImaiger A1 light microscope (Carl Zeiss, Germany) using the AxioVision 4.7.1.0 image analysis program (Carl Zeiss). ORO staining density was determined using ImageJ software (USA).

### Enzyme-linked immunosorbent assay (ELISA)

Individual (for every mouse) brain tissue was homogenized with a 15-mg sample in IS007 lysis buffer (Cloud-Clone Corp., USA) in a volume of 300 µL. The homogenates were centrifuged, and the supernatants were collected. Total 5-HT concentrations were measured using a CEA808Ge 5-HT ELISA kit (Cloud-Clone Corp.) according to the manufacturer’s instructions. The experiment was performed in triplicate.

### RNA extraction, reverse transcription, and polymerase chain reaction (PCR)

The brain was rapidly removed from each mouse and immediately frozen in a tube maintained on dry ice. Total cell RNA from brain samples was extracted using the “RNA-Extran kit” (Syntol, Russia) according to the manufacturer’s instructions and quantified by spectrophotometric analysis (ND-100F, Miulab, China). The integrity of total RNA was assessed using 1.5% agarose gels through electrophoresis and ultraviolet-visible spectrophotometry. The complementary DNA was synthesized by reverse transcription from 1 µg of total RNA using the “Reverse Transcription” reagent kit (Syntol). Real-time PCR was performed using Eva Green master mix (Syntol) according to the manufacturer’s instructions. The thermal profiles were as follows: One cycle of 2.5 min at 95°C, followed by 40 cycles of 10 s at 95°C, 20 s at 60°C, and 10 s at 72°C. Amplification was performed using DTLite4 S1 (DNA technology, Russia). Messenger RNA (mRNA) expression versus a housekeeping gene, β-actin (ACTB) was calculated using the ΔΔCt method. The primer sequences are listed in Table 2.

**Table 2:** Sequences of forward and reverse primers.

Gene	Forward primer	Reverse primer
SERT	TGCCTTTTATATCGCCTCCTAC	AGTTGCCAGTGTTCCAAGA
BDNF	AAGTTCCCCAGCGGTCTTCC	TCCAATTTGCACGCCGATCC
TPH 2	CATTCCTCGCACAATTCCAGTCG	AGTCTACATCCATCCCAACTGCTG
HTR1a	GACAGGCGGCAACGATACT	CCAAGGAGCCGATGAGATAGTT
HTR2a	TAATGCAATTAGGTGACGACTCG	GCAGGAGAGGTTGGTTCTGTTT
HTR2c	CTTCCGTATTCCCTCCCTTCCT	GCATCATTCTGGTCTCCTGCAA
ACTB	GCAAGTACTCTGTGTGGATCGG	GTAACAGTCCGCCTAGAAGCAC

### Statistical analysis

Statistical analysis was conducted using Statistica 10.0 software (StatSoft, USA) and complemented by GraphPad Prism 8.0 (GraphPad Software Inc., USA) for advanced modeling and visualization. Descriptive statistics were reported as the mean ± standard deviation for variables with a normal distribution and as the median (ME) with interquartile ranges [P25–P75] for non-normally distributed data. Categorical data were presented as percentages or absolute counts.

A two-way analysis of variance (ANOVA) was performed to evaluate the interaction effects of genotype (WT vs. HET) and treatment (Control vs. Trp) on continuous outcomes. When significant interactions or main effects were detected, *post-hoc* analyses using Tukey’s HSD or Bonferroni correction were applied to compare groups. For variables that did not meet the assumptions of normality or homogeneity of variance, non-parametric tests such as the Mann–Whitney U-test were used for independent group comparisons, and the Wilcoxon signed-rank test was employed for paired comparisons.

For repeated measures data, such as body composition and behavioral parameters over time, linear mixed models (LMMs) were recommended to account for within-subject correlations, with genotype and treatment as fixed effects, time as a repeated measure, and a random intercept for individual mice. Where applicable, the Greenhouse-Geisser correction was applied to adjust for violations of sphericity.

Correlations between continuous variables, such as biochemical parameters and behavioral outcomes, were assessed using Pearson’s or Spearman’s correlation coefficients, depending on the data distribution. Effect sizes (e.g., partial eta-squared for ANOVA, Cohen’s d for pairwise comparisons, or Spearman’s rho for correlations) were reported to contextualize the magnitude of findings.

To control for multiple comparisons, the Benjamini-Hochberg procedure was employed to limit the false discovery rate (FDR) in exploratory analyses. A critical level of significance (p < 0.05) was adopted for all analyses, with adjusted thresholds applied for *post-hoc* and multiple comparison tests.

## RESULTS

### Body composition analysis and body indexes

A two-way ANOVA revealed that there was no interaction between genotype, Trp treatment, and growth rate (F3.16 = 0.586; p = 0.63); this also applies to the Lee index (F3.16 = 0.690; p = 0.57). A significant difference was noted between the WT + Trp and HET + Trp groups: In the latter group, the growth rate was reduced by 27.3% (p < 0.05). A dependent comparison showed that the Lee index was significantly increased in WT mice by 4.7% (p < 0.05) and in HET mice by 10.1% (p = 0.02) in comparison to day 0 (Table 3).

**Table 3:** Parametric indicators of the body condition of mice.

Group	Growth rate (Rg)	Lee index		

		0 days	67 days	p-value (#, 0 vs. 67)
WT	0.004 (0.010–0.039)	309.8 (298.4–318.2)	**314.1 (321.8–341.8)** ^#^	**0.05**
WT + Trp	−0.043 (−0.054–0.001)	317.6 (306.9–340.1)	320.9 (303.9–324.1)	0.75
HET	0.016 (−0.21–0.030)	301.5 (289.2–313.1)	**326.5 (314.8–338.3)** ^#^	**0.02**
HET + Trp	−0.007 (−0.024–0.010)*	309.8 (299.3–315.7)	312.7 (310.7–328.1)	0.74

WT=Wild-type, Trp=Tryptophan, HET=Heterozygous TPH 2, The values are highlighted in bold with statistically significant differences, #p < 0.05 denotes a significant difference comparison of dependent in-group averages, the Wilcoxon test, *p < 0.05 denotes a significant difference between the two groups independent comparison, the Mann–Whitney test

**Figure 2:**
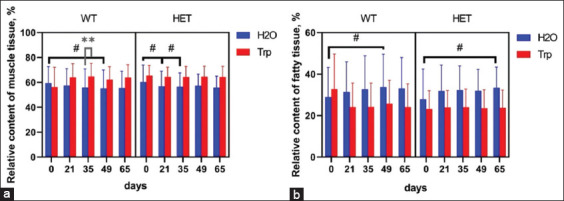
Indicators of the NMR relaxometer in experimental groups. (a) Relative content of fatty tissue; (b) Relative content of muscle tissue (*p < 0.05 denotes a significant difference between the two groups – independent comparison, the Mann–Whitney U-test; ^#^p < 0.05 denotes a significant difference comparison of dependent in-group averages, the Wilcoxon test).

It was revealed that there was no interaction between genotype, Trp treatment, and the relative content of muscle (on day 65 F3.16 = 1.453; p = 0.26) and adipose (on day 65 F3.16 = 1.730; p = 0.20) tissue. As shown in Figures 2a and b, the relative adipose tissue content in WT and HET mice tended to increase throughout the study, with a slight decrease on day 65. In contrast, the relative content of muscle tissue decreased to 9.4% by day 65 (p = 0.11) in WT mice and up to 12.0% (p = 0.06) in HET mice. WT + Trp and HET + Trp mice showed a trend toward a decrease in the relative content of adipose tissue (22.4% and 5.9%, p > 0.05) and an increase in muscle tissue throughout the study (5.9% and 2.9%, p > 0.05).

### Behavioral testing

**Figure 3:**
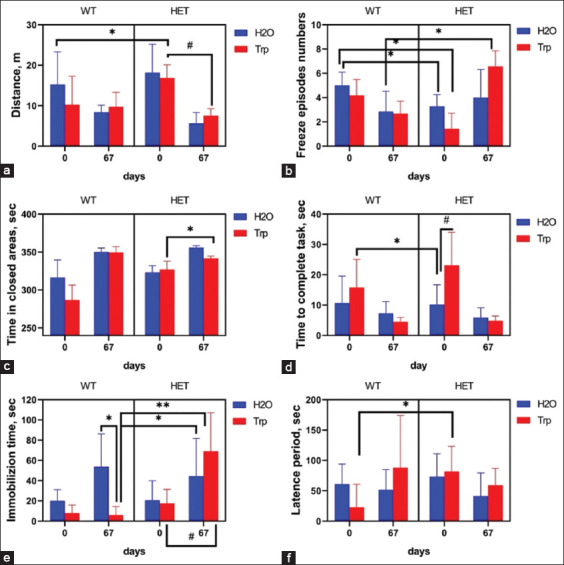
Results of behavioral tests in mice on days 0 and 67. (a) Distance covered in the test “Open field”; (b) Number of freezing episodes in the test “Open field”; (c) Time spent in closed areas in the Elevated Zero Maze; (d) Time to complete the task in “Extrapolation escape task”; (e) Time of immobilization in forced swim test; (f) Latent time of immobilization in forced swim test (*p < 0.05 denotes a significant difference between the two groups – independent comparison, the Mann–Whitney U-test; ^#^p < 0.05 denotes a significant difference comparison of dependent in-group averages, the Wilcoxon test).

In the behavioral test, “Open Field,” freezing episodes were found to be dependent on an interaction between genotype and Trp treatment (F3.16 = 6.961; p < 0.01, two-way ANOVA). There was a decrease in the distance traveled in the testing arena among all groups, so the difference for WT mice was 48.7% (p = 0.11), for WT + Trp −25.5% (p = 0.60), for HET 64.7% (p = 0.18), for HET + Trp 55.7% (p = 0.03) on day 67 relative to day 0 (Figure 3a). Less distance indicates a decrease in the anxiety of mice by the end of the experiment; in HET and HET + Trp mice, the effect was more obvious. There was a decrease in the number of freezing episodes on day 67 relative to day 0 in WT mice (by 50.0% at p < 0.05). In HET mice and HET + Trp mice, the number of freezing episodes was increased on day 67 relative to day 0 by 80.0% (p = 0.53) and 63.3% (p = 0.03) (Figure 3b). The number of freezing episodes in HET + Trp mice was 58.3% (p = 0.04) and 66.7% (p = 0.04) and WT + Trp mice by 66.7% (p = 0.03).

In the Elevated Zero Maze group, both latency period and time in closed areas were not dependent on genotype and Trp treatment (F3.16 = 2.443; p = 0.10 and F3.16 = 0.931; p = 0.45, respectively, two-way ANOVA). There was an increase in the time spent in the closed areas of the arena in mice of all groups; however, it is worth noting a decrease in this indicator in HET + Trp mice compared with HET mice by 4.5% (p < 0.05). In addition, 57% of the HET mice did not leave their location throughout the testing (Figure 3c).

In the behavioral test “Extrapolation escape task” time was not dependent on genotype and Trp treatment (F3.16 = 1.190; p = 0.34, two-way ANOVA). In mice of all groups, on day 67, the time to complete the task decreased compared with day 0: in WT mice by 31.1% (p = 0.68), in WT + Trp mice by 71.5% (p = 0.22), in HET mice by 42.2% (p = 0.45), and in HET + Trp mice by 79.2% (p = 0.02) (Figure 3d).

In the forced swim test, the total immobilization time and latency period were not dependent on genotype or Trp treatment (F3.16 = 1.202; p = 0.34 and F3.16 = 2.252; p = 0.12, respectively, two-way ANOVA). *Post hoc* testing revealed a significant increase in the total immobilization time in HET + Trp mice compared with WT + Trp mice (p = 0.03, Bonferroni test). In HET + Trp mice, an increase in immobilization time was noted by 75.8% (p < 0.05) (Figure 3e), and a latent period of 28.0% (p = 0.40) (Figure 3f) was observed on day 67 compared with the values obtained on day 0. The parameters of WT + Trp mice did not differ between tests.

### Cytometric and biochemical blood tests

**Figure 4:**
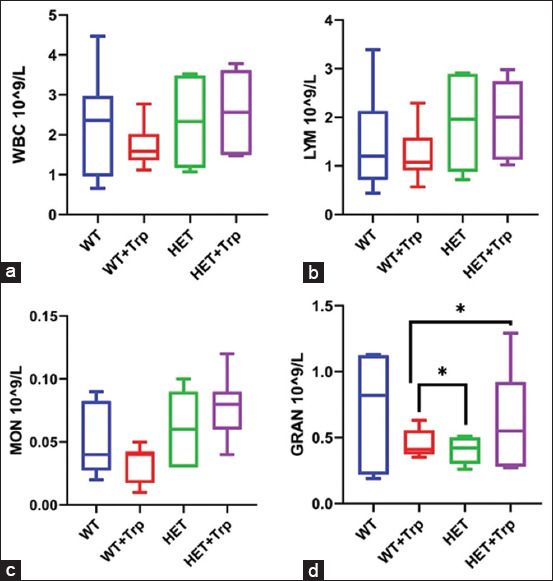
(a–d) Cytometric blood parameters in mice at the end of experiment. *p < 0.05 denotes a significant difference between the two groups (independent comparison (the Mann–Whitney U-test).

Two-way ANOVA showed an interaction between genotype, Trp treatment, and monocyte content (F3.16 = 3.010; p = 0.04). In the blood of WT + Trp mice, there was a tendency toward a lower number of white blood cells due to a decrease in the number of MON (relative to HET + Trp mice by 50% at p = 0.01; relative to HET mice by 35.9%, p < 0.05) (Figure 4).

The influence of genotype and Trp treatment on sodium and triglyceride content was revealed (F3.16 = 4.433, p = 0.02 and F3.16 = 3.311, p < 0.01). *Post hoc* test revealed an increase in triglyceride content in HET mice relative to WT mice (p < 0.01, Bonferroni test) and a decrease in HET + Trp mice relative to HET mice (p < 0.01, Bonferroni test). In the blood plasma of WT + Trp mice compared with WT mice, a decrease in the urea content by 9.3% (p < 0.01) was noted; in comparison with HET mice, an increase in the concentration of albumin by 2.7% (p = 0.03), and triglycerides by 12.2% (p < 0.05), a decrease in the urea content by 9.3% (p = 0.03), and cholesterol by 11.3% (p = 0.03) was noted (Table 4).

**Table 4:** Biochemical analysis of blood plasma at the end of experiment. Data are presented as median and interquartile range (P25–P75).

Parameter	WT	WT + Trp	HET	HET + Trp
Sodium (mmol/L)	124.05 (114.83–130.46)	123.88 (119.45–130.51)	123.50 (118.2–125.7)	**129.30^f^ (124.7–130.3)**
Glucose (mmol/L)	28.80 (27.83–29.85)	**25.15^a^ (23.95–25.75)**	26.40 (24.80–29.70)	**22.70^f,c^ (21.70–24.75)**
Total protein (g/L)	49.55 (46.60–50.18)	48.05 (45.98–49.45)	45.70 (45.50–47.05)	**44.20^f,c,e^ (42.45–44.88)**
Albumin (g/L)	21.65 (20.95–22.73)	**22.15^d^ (21.68–22.55)**	**23.50^b^ (22.85–23.75)**	22.60 (21.85–23.5)
Creatinine (μmol/L)	57.30 (55.78–58.83)	56.15 (55.70–56.71)	56.10 (52.95–61.05)	**52.20^c^ (52.15–55.10)**
Urea (mmol/L)	8.55 (8.50–8.68)	**7.75^a,d^ (7.55–7.80)**	8.10 (8.05–8.70)	**7.60^f,c^ (7.35–7.85)**
Triglycerides (mmol/L)	0.65 (0.52–0.70)	**0.74^d^ (0.62–0.89)**	**1.04^b^ (0.88–1.19)**	**0.44^f^ (0.43–0.64)**
Total cholesterol level (mmol/L)	2.55 (2.23–2.75)	**2.26^d^ (2.13–2.48)**	**3.03^b^ (2.95–3.21)**	**2.84^f^ (2.44–2.98)**
LDL (mmol/L)	0.16 (0.15–0.19)	0.16 (0.14–0.17)	0.18 (0.18–0.19)	**0.15^f^ (0.14–0.17)**
HDL (mmol/L)	1.15 (1.03–1.28)	1.04 (0.99–1.12)	1.24 (1.11–1.31)	**0.99^f^ (0.92–1.13)**
AI (r.u.)	1.21 (1.13–1.17)	1.16 (1.15–1.24)	**1.45^b,d^ (1.39–1.74)**	**1.86^c,e^ (1.44–1.60)**
De Ritis ratio (r.u.)	2.31 (2.87–4.29)	**3.85^d^ (4.44–4.78)**	3.61 (3.62–4.05)	**3.76^c,e^ (3.47–4.70)**

The values are highlighted in bold with statistically significant differences, p < 0.05 between the two groups (the Mann-Whitney U-test): aWT and WT + Trp, bWT and HET, cWT and HET + Trp, dWT + Trp and HET, eWT+Trp and HET + Trp, fHET and HET + Trp . LDL=low-density lipoproteins, HDL=High-density lipoproteins, AI=Atherogenic index, r.u=Relative units, WT=Wild-type, Trp=Tryptophan, HET=Heterozygous TPH 2

In HET mice relative to WT mice, an increase in the triglyceride concentration was observed by 37.5% (p < 0.01) was revealed.

In the blood plasma of HET + Trp mice relative to HET mice, an increase in sodium concentration by 4.5% (p = 0.05), a decrease in protein content by 3.3% (p < 0.05), and glucose by 16.6% (p < 0.05), urea by 6.2% (p < 0.05), triglycerides by 57.6% (p < 0.05), LDL by 16.7% (p = 0.07) and HDL by 20.1% (p < 0.05) was revealed.

A two-way ANOVA revealed that there was an interaction between genotype, Trp treatment, and the de Ritis coefficient (F3.16 = 5.132; p = 0.01); this also applies to AI (F3.16 = 4.084; p = 0.02).

An interesting pattern was noted between the groups: in WT + Trp mice, the protein concentration and the de Ritis coefficient exceeded the values of HET + Trp mice by 8.0% (p = 0.03) and 2.3% (p = 0.02). The de Ritis coefficient of WT + Trp mice was increased by 30.0% (p < 0.01), relative HET mice; in WT + Trp mice and HET + Trp mice in relation to WT mice, the coefficient was increased by 66.7% (p < 0.05) and 62.8% (p > 0.05). In HET and HET+Trp mice, the AI was higher than that in WT mice by 19.8% and 53.8% (p < 0.05) and higher than that in WT + Trp mice by 25.0% and 60.3% (p < 0.05).

We observed no statistically significant differences in the concentrations of low-density lipoproteins in blood plasma between the comparison groups (Table 3).

### Internal organ study

**Figure 5:**
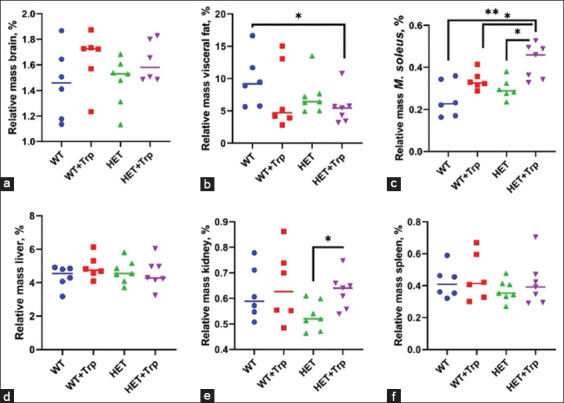
The relative masses of the internal organs of animals. (a) Brain, (b) Visceral fat, (c) *M. soleus*, (d) Liver, (e) Kidneys, and (f) Spleen (*p < 0.05 and **p = 0.01 independent comparison of groups, the Mann–Whitney U-test).

A two-way ANOVA revealed that there was no interaction between genotype, Trp treatment, and the internal organs of mice besides the relative mass of *M. soleus* (F3.16 = 4.231; p = 0.02). In HET + Trp mice was revealed: A decrease in the relative mass of visceral fat by 40.5% (p = 0.04) in comparison to WT mice; an increase in the relative mass of *M. soleus* by 50.7% (p = 0.02) in comparison to WT mice, by 28.9% (p = 0.04) in comparison to WT + Trp mice, and by 40.9% (p = 0.01) in comparison to HET mice; and an increase in the relative mass of the kidney by 23.2% (p = 0.02) in comparison to HET mice (Figure 5).

The relative weight of the liver did not differ significantly between the groups, whereas the highest values were observed for WT + Trp mice (by 4.2% relative to WT mice and HET mice; by 9.9% relative to HET + Trp mice).

**Figure 6:**
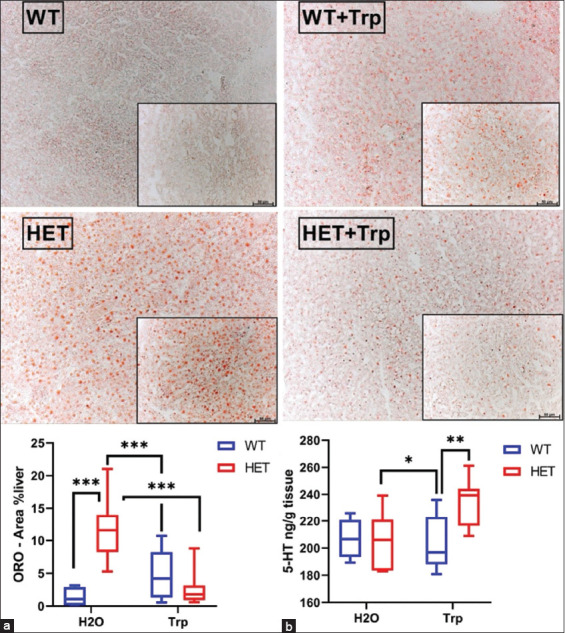
A representative section for each group and Oil-red O staining of liver lipid levels. (a) Size of lipid droplets in the liver of mice stained with ORO (***p = 0.005 independent comparison of groups, the Mann–Whitney U-test). (b) 5-hydroxytryptamine content in the brain. (*p < 0.05 and **p < 0.01 are independent comparisons of groups, the Mann–Whitney U-test).

The influence of genotype and Trp treatment on ORO staining of liver tissue was revealed (F3.16 = 7.742, p < 0.01, two-way ANOVA). The results of the histological study did not reveal an increase in the staining area of ORO fat droplets in the *M. soleus* tissues of mice of all groups. ORO staining of liver tissues revealed that HET mice had larger lipid droplets and a larger staining area compared with HET + Trp mice by 4.9 times (p < 0.01), as well as WT mice (by 6.9 times p < 0.01) and WT + Trp mice (by 2.4 times, by p < 0.01) (Figure 6). It is also worth noting the increase in the size of lipid droplets and in the ORO staining of the livers of WT + Trp mice relative to WT mice by 2.9 times and relative to HET + Trp mice by 2,0 times (p > 0.05) (Figure 6a).

The influence of genotype and Trp treatment on 5-HT concentration in mouse brains was revealed (F3.16 = 7.640, p = 0.01, two-way ANOVA); 5-HT content increased in the brain of HET + Trp mice relative to WT + Trp mice by 23.9% (p < 0.01, Bonferroni test). Interestingly, the concentration of 5-HT in the brains of WT + Trp mice was also lower than that of HET mice by 17.5% (p = 0.03) (Figure 6b).

### mRNA expression assessment of serotonergic genes

**Figure 7:**
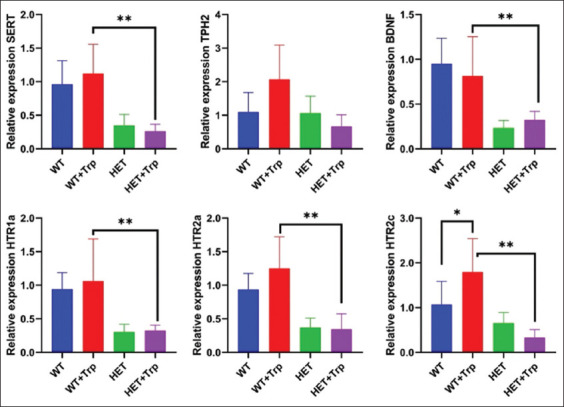
Analysis of messenger RNA expression levels of serotonergic genes and *BDNF* (***p < 0.0001, **p < 0.001, *p < 0.05).

In the brain tissues, the influence of the “genotype” factor on the relative gene expression of the following genes was noted: *SERT* (F_3,10_ = 20.57; p = 0.001), *HTR1a* (F_3,10_ = 21.20; p < 0.001), *HTR2a* (F_3,10_ = 26.85; p < 0.001) and *BDNF* (F_3,10_ = 26.40; p < 0.001), while the changes were characteristic for both control animal groups and those consuming Trp (Figure 7). *SERT* gene expression was reduced in the WT group relative to HET by 63.54% (p < 0.01), in the WT + Trp group relative to HET + Trp by 76.78% (p < 0.01); The *HTR1a* gene was reduced in the WT group relative to HET by 68.08% (p < 0.01), in the WT + Trp group relative to HET + Trp by 69.81% (p < 0.01); the *HTR2a* gene was reduced in the WT group relative to HET by 60.63% (p < 0.001), in the WT + Trp group relative to HET + Trp by 72.79% (p < 0.001); the *BDNF* gene was reduced in the WT group relative to HET by 75.78% (p < 0.001), in the WT+Trp group relative to HET + Trp by 60.49% (p < 0.01). Moreover, only in the WT groups, relative to HET + Trp, Trp consumption contributed to an increase in gene expression only for *HTR2c* by 68.22% (F = 19.88; p < 0.05). For the same gene, the influence of the “genotype + supplement” factors on the gene expression index was determined (F = 6.37; p = 0.02).

Analysis of relative gene expression changes according to genotype and Trp consumption revealed that the most pronounced relative expression of serotonin-related genes was observed in the WT + Trp group, with a decrease in *BDNF* expression, relative to the WT group. In TPH2 HET + Trp animals, an increase in *BDNF* expression was observed, with a decrease in genes associated with the implementation of serotonin in neurons, with the exception of serotonin receptors (*HTR1a* and *HTR2a*), relative to the TPH2 HET control group.

## DISCUSSION

Trp metabolism has emerged as a central hub for the metabolic control of immunological and neuropsychological processes [38], whereas the metabolic pathways of Trp depend on both age and the genetic component of the organism. In our study, we revealed interesting facts about the effect of Trp on the anabolic processes and behavioral and cognitive performance of aging mice, which are prone to depression, but without severe symptoms of depression, compared with WT mice.

Trp can be considered a building block for muscle mass growth, and dietary amino acid intake is the single most important dietary variable that increases the rate of muscle protein synthesis, which was considered by Dukes *et al*. [39] regarding dietary manipulations with specific amino acids that have been considered *in vivo* [40]. We revealed in WT + Trp mice a decrease in body weight: At a constant Lee index, the growth rate significantly decreased relative to that of WT mice. There was also a tendency to increase the relative muscle mass (according to the results of NMR relaxometry) and the relative mass of *M. soleus*, whereas the relative content of adipose tissue, it was noted to decrease. These data were consistent with biochemical analysis, which revealed high plasma protein levels in WT mice relative to HET + Trp mice.

In a study by Priatno *et al*. [41], it was found that Trp supplementation in the diet of cattle (Hanwoo bulls) resulted in an increase in the expression of genes associated with lipid catabolism and fatty acid transport into muscle tissue. In our study, we observed an increase in the size of lipid droplets and the staining area of the liver of WT + Trp mice relative to WT and HET + Trp mice, with an insignificant decrease in high-density lipoproteins, cholesterol, and triglycerides relative to WT mice. In addition, regarding WT + Trp mice with high de Ritis coefficient values, a significant decrease in the level of urea was observed.

The described conditions are supplemented by reduced monocyte content, in combination with the fact that 80%–90% of Trp are degraded by the indole and kynurenine pathways, are modulated by inflammatory mediators [42], and may be accompanied by impaired production and release of insulin [43]. These data are interesting because aging women often experience immunosenescence associated with an increase in the content of MON and their activation [44, 45]. When analyzing behavioral tests in WT + Trp mice, a decrease in immobility was observed. In a previous study by Gmoshinski *et al*. [46], Trp reduced the time required for immobility and aggressive behavior in mice. Such an increase in motor activity in mice may be due to an increase in the transport of exogenous Trp to the brain with subsequent changes in the noradrenergic and serotonergic activities of neurons [47].

In our study, an obese phenotype was identified for female HET mice, prone to depression, aged >1 year: Lee index increased, the content of muscle tissue decreased, high values of total cholesterol, lipoproteins, and triglycerides were found in blood plasma, and accumulations of lipids in the liver. Similar changes were reported in [48] against the background of depression-like behavior in 12-month-old homozygous 5-HT-deficient female mice. The mice did not exhibit pronounced depression-like behavior during the study because HET mice are considered to be endophenotypically normal [49] and had brain 5-HT levels similar to those of WT mice. However, mice preferred to be in protected areas and showed reduced innate motivation to explore new environments in the O-maze test.

In HET + Trp mice, the concentration of 5-HT in the brain was increased relative to that in WT + Trp mice, which may indicate a more efficient synthesis pathway within the 5-HT system and an increase in the action of selective 5-HT reuptake inhibitors [50]. At the same time, in HET + Trp mice, a decrease in the time in the closed arms of elevated Zero Maze was not related to HET mice. The increased exploration of open arms can be interpreted as a low level of anxiety [51]. In our study, HET + Trp mice showed increased numbers of freezing in the Open Field test and immobilization time in the forced swim test compared with WT + Trp, such interesting changes support the well-established facts about a steady decrease in locomotion and immobility in “sedative” or “antidepressant” effect of Trp [52–55]. Potentially, Trp can enhance memory and attention [56], on the basis of which we can conclude that Trp treatment has a positive effect on cognitive functions, which we confirmed with the “Extrapolation Escape Task” test.

Trp improves memory in older rats [57] and improves sleep/wake cycles in older people [58]. In addition, as in our study, Trp reduced blood glucose levels and delayed the progression of diseases associated with aging [59].

In the present study, we demonstrated that HET + Trp mice are protected from fatty infiltration of the liver: The liver ORO staining area decreased relative to that of HET and WT + Trp mice. At the same time, we observed high values of total cholesterol and AI of blood plasma, with a decrease in triglycerides, low-density lipoproteins, and high-density lipoproteins; and a high level of cholesterol relative to HET mice.

The results of gene expression assessment in WT + Trp group mice showed an increase in the expression of serotonergic genes (*SERT*, *TPH2*, *HTR1a*, *HTR2a*, and *HTR2c*), whereas no changes in the expression of the *BDNF* gene were found, indicating a positive effect of Trp consumption. In the HET + Trp group, a tendency toward a decrease in the expression of genes involved in the regulation of serotonergic neurotransmission was noted relative to the HET group, whereas the expression of the *BDNF* gene was higher, indicating an increase in neural connections.

These data suggest that Trp administered to HET mice is converted by microbiota bacteria into the Trp metabolite indole, which regulates lipid catabolism. This assumption was confirmed in a previous study by Gmoshinski *et al*. [46], which showed the hepatoprotective properties of a low physiological dose of indole in a genetic model of obesity and associated nonalcoholic fatty liver disease without improving steatosis and lipid homeostasis. It should be noted that the literature contains conflicting data on the effects of the bacterial metabolite of Trp, indole, on lipid metabolism and the behavior of rats. Thus, Trp in the rat diet caused a decrease in cognitive functions and increased the severity of anxiety-like behavior. The body weight of such animals decreased, which was explained by the high search activity and high muscle tone of the rats, and indole metabolites of Trp explained the increase in fatty degeneration of the liver [60].

Trp metabolism through the kurenin pathway was similar in WT mice, as evidenced by the observed anabolic effects. Thus, the growth rate of HET + Trp mice was reduced with a decrease in the relative content of adipose tissue and an increase in muscle mass (according to the results of NMR relaxometry) as well as in the relative mass of *M. soleus* up, relative to HET mice.

The effects of Trp on WT and HET mice prone to depression indicate a multidirectional effect of Trp metabolites on systemic homeostasis at the molecular level. As we expected, the administration of Trp had a stimulating effect on the production of 5-HT in the brain, but not in WT mice, but not WT mice. The study of metabolites corresponding to different pathways of Trp metabolism is essential and will be undertaken in future studies. A more detailed study of the various modes of Trp metabolism using a knockout mouse model will expand our understanding of the functions of Trp and its metabolites for use in neuro-nutrition, especially for patients prone to depression and those who have experienced depression. This is especially true in view of the growing awareness in humans that Trp supplements and foods rich in Trp do not fulfill their promise [61].

Our hypothesis was single nucleotide polymorphism, which is characteristic of HET mice, which puts mice at risk of developing mental disorders and alters Trp pathways toward the indole and serotonergic pathways, which requires investigation.

## CONCLUSION

This study demonstrates that Trp supplementation exerts significant genotype-specific effects on aging female mice, influencing their physiology, behavior, and molecular profiles. HET mice exhibited improved cognitive performance, reduced anxiety-like behaviors, and hepatoprotective effects following Trp supplementation, alongside increased brain serotonin levels and upregulated BDNF expression, indicative of enhanced neuroplasticity. In contrast, Trp-treated WT mice displayed increased muscle growth and reduced adiposity but also a rise in liver lipid accumulation, reflecting potential metabolic trade-offs.

A key strength of this study is the comprehensive approach that integrated behavioral, physiological, biochemical, and molecular assessments over a prolonged experimental period. This allowed for a robust exploration of Trp’s effects on neuropsychiatric and metabolic outcomes in aging mice, particularly in the context of genetic susceptibility to depression.

However, the study is limited by the small sample size, which may constrain the generalizability of the findings. In addition, while the focus on female mice addresses a critical gap in research, it precludes conclusions about sex-specific responses. The study also did not explore region-specific brain neurotransmitter dynamics or the interplay between Trp metabolism and gut microbiota.

Future research should aim to expand sample sizes, include male mice, and explore sex- and age-specific effects of Trp. Investigating the gut-brain axis, region-specific brain changes, and the role of Trp metabolites in regulating lipid metabolism and behavior will provide deeper insights. Furthermore, studying Trp’s impact in human models with genetic predispositions to depression could help translate these findings into tailored dietary or therapeutic interventions.

This study underscores the therapeutic potential of Trp as a dietary intervention, particularly for individuals genetically predisposed to depressive disorders or age-related metabolic dysfunctions. However, its application should be carefully personalized to mitigate potential metabolic risks and optimize its benefits.

## AUTHORS’ CONTRIBUTIONS

EV: Conceptualization. EV and GT: Data curation. AK, EV, EK, EP, VP, and SK: Formal analysis. LF: Project administration. AK, GT, and SK: Investigation. AK, EK, EP, VP, and SK: Methodology. EV and LF: Supervision and writing, reviewing, and editing. AK and VP: Visualization. AK and EK: Writing the original draft. All authors have read and approved the final manuscript.
